# Antioxidant Capacity of Anthocyanins and other Vegetal Pigments

**DOI:** 10.3390/antiox9080665

**Published:** 2020-07-25

**Authors:** Noelia Tena, Agustin G. Asuero

**Affiliations:** Department of Analytical Chemistry, Faculty of Pharmacy, The University of Seville, 41012 Seville, Spain; ntena@us.es

Anthocyanins are the largest group of phenolic pigments, being effective hydrogen donors. The term came from the Greek anthos (flower) and kyanos (blue), proposed in 1835 by the German pharmacist L.C. Marquart to name the blue pigments of flowers. However, not only blue, but also virtually all the red, blue, and violet hues of flowers, fruits, stems, leaves, and roots correspond to pigments of this kind [1].

Fruits and vegetables are a source of a variety of micronutrients including minerals, fibres, and vitamins, and a series of compounds named phytochemicals, among which anthocyanins [2] of hydrophilic nature, and carotenoids and chlorophylls [3] of lipophilic character, are found. These plant pigments attracted great attention initially due to their physiological importance for plants, and then, due to their capacity to capture free radicals, thus, increasing antioxidant activity and preventing cell oxidation, among other biological effects. However, nowadays, the pigment compositions of some fruits, vegetables, and flowers are not completely known yet, despite the developments produced in chemical science and technology in recent decades. More recently, different papers have addressed the attention to ascertain the association between the colour of fruits and vegetables and their total antioxidant capacity [4].

This Special Issue of *Antioxidants* contains six contributions—three research articles and three reviews—including recent advances on the matter. They deal with the structure, composition, and amount of anthocyanins, carotenoids, and chlorophylls pigments in different vegetal sources. This issue also shows the action mechanisms of these kinds of antioxidants in health and disease, showing them from different approaches and points of view. In addition, some innovative applications of these compounds, proving their broad-based range of benefits for human health, are presented. To the authors who have made this Special Issue possible with their brilliant contributions, we are very grateful. We sincerely believe this topic was worth it. Many thanks are given to *Antioxidants* for the opportunity we have had to act as editors in this Special Issue. The reviewers have also carried out an important task worthy of mention and praise.

The most common antioxidant methods applied to determine the antioxidant capacity of anthocyanins and other vegetal pigments have been reviewed by Noelia Tena et al. [2] and María Roca et al. [3]. There are a variety of methods to measure antioxidant activity, either in vitro or in vivo, or a combination of both. All antioxidant determination methods have advantages and disadvantages and their correlation with biological activity in vivo is very difficult, if any at all, in all of the cases. It is a complex issue and deciding in favour of one or the other method depends, in many cases, on personal preferences.

Senejoux et al. [5] report on the potent antiglycoxidant power, e.g., by the DPPH method, and health benefits derived from bilberries consumption through antiglycoxidant anthocyanins studies from *Vaccinium myrtillus* fruit. A methylglyoxal precolumn HPLC method easily allows the identifying of the main species responsible for radical and carbonyl trapping effects; the I_50_ values of pure anthocyanin glucosides that exert the most effective radical scavenging activity were calculated. However, to provide information about the physiological relevance of these activities, it is necessary to carry out in vivo assays.

Scarce studies have been carried out on the protective effects of anthocyanins incorporated into matrices for topical delivery. The results obtained by Monica Giusti et al. [6] on this direction are very interesting in both the pharmaceutical and cosmetic industries, by showing anthocyanins to be beneficial ingredients in skin care products. The necessary requirements to exert a proper biological activity by a given topical formulation are briefly stressed by the authors. Keeping this basis in mind, single experiments with minimal sample preparation are planned, avoiding unnecessary complexity in this respect. The powerful ATR-FTIR analytical technique affords a great number of experimental data, which may be clearly interpreted with the help of the relevant partial least squares chemometrics tool.

An important study carried out by Ana M. Troncoso and Cătunescu et al. [7], dealing with the strong antioxidant activity and potential antibacterial capacity of specific anthocyanins from blueberries cultivated in a hot climate, has been included in this issue, proving the beneficial effect of these compounds in specific applications in both in vitro and in vivo assays. The results of these analyses have demonstrated the strain specificity in urinary tract infections (UTIs) of these compounds. The blueberries anthocyanins present a specific effect against *P. aeruginosa* and *K. pneumonia* ssp. *pneumoniae*. These results pointed out that these compounds could be a potential solution to avoid the antibiotic resistant detected in the effectiveness for the treatment of UITs.

Nature is the best available laboratory. Given the complexities associated with obtaining remedies via chemical synthesis, traditional medicine is sometimes a useful alternative. It is, thus, possible to obtain active ingredients from the plant material itself, or to use isolated principles from them as a starting point for obtaining complex molecules with therapeutic interest, thus, saving a large number of steps in its synthesis, and, therefore, reducing the cost of the final product. 

The study of the medicinal properties of plants in any of its various aspects might be considered today as a hot topic. The merit of the contribution of Chavez-Servia et al. [8] is to bring all edible leafy plants from Mexico together in a review, with the purpose of studying their nutritional, nutraceutical, and antimicrobial potential, thus, filling a gap in the bibliography. The wide spectrum of the journals cited is an additional value of the contribution, in which the potential effect of new or exotic sources of antioxidants, less well known, has been clearly documented. 

The review of María Roca et al. [3] discusses different approaches used for carotenoids and chlorophylls to draw a physiological perspective, from in vitro studies to in vivo assays, by using oxidative biomarkers. Many studies have shown how a pigment’s structure influences the antioxidant response and underlying mechanism; this knowledge is vital to understanding new data in a metabolic networks context, making possible direct applications to human health.

The pharmaceutical Achilles heel of anthocyanins is their existence as various molecular forms in equilibrium, depending widely on temperature, pH, the presence of light and oxygen. Anthocyanins show complex biochemistry and there is still much to be discovered about their biochemical activity and clinical pharmacology. As evidenced by the therapeutic effects of phytochemicals, it is important to understand the nature of absorption and metabolism “in vivo”, since this knowledge will allow the development of new food products, both fresh and manufactured, with greater therapeutic efficacy, as noted by Noelia Tena et al. [2].

In this regards, the food enriched in this kind of pigment is appreciated due to their health properties. In order to ensure the beneficial effects of these products, the U.S. Department of Agriculture has published a database that compiles the total concentration of anthocyanins in different fruits and vegetables together with the daily intake necessary in each case to obtain a positive effect in the human health [9]. Furthermore, currently, the European Food Safety Authority (EFSA) has published guidance for the scientific requirements for health claims related to antioxidants in foods and their implication in cardiovascular health [10]. 

In this Special Issue, the individual composition and concentration of anthocyanins, carotenoids, and chlorophylls in different fruits, vegetables, and flowers have been reported [2,3]. A better knowledge is acknowledged of antioxidant mechanisms of action of anthocyanins, carotenoids, and chlorophylls pigments. The issue also provides information about different ex vivo and in vivo studies that prove the key role of these compounds in the prevention and/or inhibition of diseases, promoting also their application as functional foods, and in the cosmetic field as a bioactive ingredient with antibacterial effect or as photoaging inhibitors. Thus, the literature suggests that anthocyanins, carotenoids, and chlorophylls may have important health effects, although the basic aspects of their absorption and metabolism are not yet clearly established. However, despite all these setbacks, the future is bright, since once the metabolism is completed and their true biological activities established, their beneficial effects on human health could be fully explored.
